# Efficacy and tolerability of an oral supplement containing amino acids, iron, selenium, and marine hydrolyzed collagen in subjects with hair loss (androgenetic alopecia, AGA or FAGA or telogen effluvium). A prospective, randomized, 3‐month, controlled, assessor‐blinded study

**DOI:** 10.1111/srt.13381

**Published:** 2023-06-05

**Authors:** Massimo Milani, Francesca Colombo, GFM‐O‐Trial Investigators Group, Chiara Baraldo, Mauro Barbareschi, Paolo Chieco, Laura Colonna, Mandel Victor Desmond, Maria Cristina Fiorucci

**Affiliations:** ^1^ Medical Department Cantabria Labs Difa Cooper Varese Italy; ^2^ Medical Department Cantabria Labs Difa Cooper Padova Italy; ^3^ Medical Department Cantabria Labs Difa Cooper Milano Italy; ^4^ Medical Department Cantabria Labs Difa Cooper Ruvo di Puglia Italy; ^5^ Medical Department Cantabria Labs Difa Cooper Roma Italy; ^6^ Medical Department Cantabria Labs Difa Cooper Modena Italy; ^7^ Medical Department Cantabria Labs Difa Cooper Genova Italy

**Keywords:** androgenic alopecia, hydrolysed fish‐origin collagen, oral supplementation, telogen effluvium

## Abstract

**Background:**

Oral supplementation with some amino acids (like methionine, taurine, and cysteine) could be useful in subjects with hair loss conditions such as androgenic alopecia (AGA or FAGA) or telogen effluvium (TE). Hydrolysed collagen (HC) oral supplementation has demonstrated to have beneficial effects on nail and skin health and could improve hair growth. A food supplement in tablet formulation containing hydrolysed fish‐origin collagen (300 mg/dose), taurine, cysteine, methionine, iron, and selenium has been recently available. To date no controlled data are available regarding the clinical efficacy of this product as adjuvant to hair loss specific treatments in these clinical conditions.

**Study aims:**

To evaluate and compare the efficacy and tolerability of an oral supplementation based on HC and amino acids in subjects with hair loss due to AGA/FAGA or chronic TE in combination with drug treatments in comparison with drug treatments alone.

**Methods and subjects:**

In a prospective, 12‐week, randomized, assessor‐blinded controlled trial 83 subjects (mean age 41 ± 16 years; 26 men and 57 women) were enrolled in the study. Fifty‐nine subjects suffered from AGA/FAGA (Hamilton I‐VA, Ludwig I‐1, II‐2) and 24 from chronic TE. Subjects were randomized to oral supplementation (1 tablet day) in combination with the specify drug treatment decided by the investigator according to the type of hair loss (AGA/FAGA or TE) (Group A; *N* = 48) or to specific drugs treatment only (Group B; *N* = 35). The main outcome of the trial was the clinical efficacy evaluation using a 7‐point global assessment score (GAS) (from +3: Much Improved to ‐3 Much worsened; with score 0 representing no modification). The GAS score was evaluated using standardized photographs by an investigator unaware of the treatment groups at week 6 and at week 12. A secondary outcome was the evaluation of acceptability of the treatment regimen using a 10‐point evaluation score.

**Results:**

Seventy‐six participants (91.6%) completed the 12‐week study period. The GAS score at week 6 was 0.5 ± 0.2 in group A and 0.0 ± 0.1 in Group B (*p* < 0.05; Mann‐Whitney). At week 12 the GAS score in Group A was statistically significant higher in comparison with Group B (1.67 ± 0.16 and 0.66 ± 0.20, *p* < 0.001; Mann–Whitney test). A higher percentage of Group A subjects achieved a GAS score of ≥2 in comparison with group B (50% vs. 23%). The oral supplement was generally well tolerated.

**Conclusion:**

An oral supplement containing hydrolysed fish‐origin collagen, taurine, cysteine, methionine, iron, and selenium has demonstrated to improve the clinical efficacy of specific anti‐hair loss treatments in subjects with AGA/FAGA or chronic TE.

## 1 INTRODUCTION

Hair loss can be classified in several forms including male‐ or female‐androgenetic alopecia (AGA and FAGA, respectively), and telogen effluvium (TE). AGA is one of the most frequent hair loss causes,^1^ affecting 50% of men by 50 years of age and nearly 50% of women.^2^ This condition is characterized by hair follicular miniaturization. TE is a common cause of diffuse nonscarring hair loss that is usually correlated by a physiological stress.^3^ Topical minoxidil and oral finasteride are the only treatments authorized for AGA.^4^ Hair loss treatments can achieve a satisfy clinical respond rate in term of hair regrowth in not more than 50%–60% of patients. For this reason, adjuvant products are commonly used in combination with drug treatments (e.g., finasteride or minoxidil) in hair loss patients with the aim to increase the responder rate.^5^ Therefore, cosmetic products or specific oral supplementations could be beneficial in subjects with hair loss conditions. Some amino acids, like methionine, taurine, and cysteine could be beneficial in subjects with hair loss conditions like AGA, FAGA, or TE. Recent studies have been underlined the role of cysteine as indicator of hair health, indeed this amino acid is able to influence the mechanisms implicate in hair loss.^6^, ^7^ Methionine is an essential amino acid that represents a key constitutive element of keratin, an important protein present in hair. An in vitro study underlined the capability of methionine to upregulate β‐catenin. The Wnt/β‐catenin signalling pathway plays an essential role during hair follicle induction.^8^ Taurine is an amino acid produce by methionine and cysteine metabolism. It is involved in different functions including immunomodulatory and antifibrotic properties. In addition, it was demonstrated that taurine promoted in vitro hair survival and prevented the deleterious effects of transforming growth factor, an inhibitor of hair growth, on hair follicle.^9^ Hydrolysed collagen (HC) oral supplementation has demonstrated to have beneficial effects on nail and skin health and could improve hair growth. Different studies have been reported the role of HC peptides in enhancing hair thickness and follicle cell proliferation, and they ability in improving hair dryness and dullness.^10^, ^11^ In recent years, fish‐derived collagen has emerged as an alternative and interesting source with bioactive properties. Indeed, fish‐derived HC presents some advantages such as low toxicity, low inflammatory responses, better absorbability, environmental friendliness, and less regulatory and quality control problems.^12^ Although there is a weak evidence supporting their use, also micronutrients could be involved in hair loss management.^13^ Iron represents an essential element involve in different human function such as red blood cells production, function of enzymes and transcription factors, and DNA synthesis.^14^ Iron supplementation can be beneficial in patients with air loss and could be evaluated case‐by‐case.^13^ Also selenium deficiencies have associated with air loss. Selenium is a trace element involved in the function of different proteins with antioxidant and anti‐inflammatory functions.^15^ A product in tablet formulation containing hydrolysed fish‐origin collagen (300 mg/dose), taurine, cysteine, methionine, iron, and selenium has been recently commercialized as food supplement for patients with hair loss disorders. However, no controlled data are available so far regarding clinical efficacy of this product as adjuvant to hair loss specific treatments in these clinical conditions.

## 2 STUDY AIM

The aim of this study was to evaluate the efficacy and tolerability of an oral supplementation (based on HC, amino acids, and micronutrients) in combination with drug treatments (e.g., minoxidil or finasteride) in subjects with hair loss due to AGA/FAGA or TE in comparison with drug treatments alone.

## 3 SUBJECTS AND METHODS

### 3.1 Population and study design

Between May 2022 and November 2022, 83 subjects with AGA, FAGA or chronic TE were assessed and screened for inclusion in the trial. Hair loss condition was confirmed by physical examination performed by a dermatologist that assigned a clinical diagnosis of AGA or chronic TE. AGA was diagnosed using the Hamilton or Ludwig scales by observing hair thinning and miniaturization in scalp region. Chronic TE was diagnosed considering subject history, self‐reported and investigator‐reported thinning hair. The study was designed as a 12‐week, prospective, randomized, assessor‐blinded trial. The trial took place in six different Dermatology clinics in Italy. The trial was conducted according to Good Clinical Practice Guidelines and Helsinki Declaration. All subjects provided signed informed consent. Eighty‐three subjects (mean age 41.2 ± 16.2 years) with AGA/FAGA (71.1%) or TE (28.9%) were enrolled. The main inclusion criteria were age >18 years, diagnosis of AGA/FAGA or chronic TE, eligible for the treatment with the oral supplement used in the study. The main exclusion criteria were acute inflammatory condition at the scalp, allergies to the components of the products used in the study, iron deficiency, clinically relevant alteration to thyroid function, presence of other scalp conditions (e.g., alopecia areata, seborrheic dermatitis, psoriasis, mycosis, lichenoid lesions). The study was conducted over 12 weeks, patients was evaluated at baseline and after 6 (facultative visit) and 12 weeks. Patients were randomly divided in two arms: Group A (*n* = 48) received 1 tablet day of the oral supplement (GFM oral, Cantabria Labs Difa Cooper, Caronno Pertusella, Italy) and a specify drug treatment decided by the investigator according to the type of hair loss (AGA/FAGA or TE); Group B (*n* = 35) received the specific drugs treatment only. Randomization list was generated by a dedicated computer program.

### 3.2 Study outcomes

The primary efficacy endpoint was evaluated in an assessor‐blinded fashion after 6 (facultative visit) and 12 weeks by a panel of six dermatologist (GFM‐O‐Trial Investigator Group) through the global assessment score (GAS), a 7‐point score from −3 (severe worsening) to +3 (excellent improvement) performed using standardized global photographs, taken with the head in a stereotactic positioning device.^16^ This technique has previously been demonstrated to have excellent test‐retest reproducibility and interrater agreement.^17^, ^18^

The second endpoints were represented by the global clinical efficacy and the global tolerability evaluated by the investigators after 12 weeks through a 5‐point score from −2 (very negative) to +2 (very positive). The global clinical efficacy was also evaluated by patients through a 5‐point score from −2 (severe worsening) to +2 (excellent improvement). The patients also evaluated the acceptability and tolerability of the products using a scale from 1 (very accepted) to 10 (not accepted).

### 3.3 Statistical analysis and sample size calculation

A total of 83 participants were enrolled and randomized in two groups. Sample size calculation was performed considering the data of Panchaprateep and Lueangarun,^19^ that reported an increase of GAS after the use of a specific drug treatment for hair loss (5 mg oral minoxidil) of +1.7 ± 0.6, and a hypothesis to find an additional effect in GAS from the combination of the supplementation with the drug treatment of about 20%. With an effect size (Choen's d value) of 0.9, an alpha value of 0.05 and a power of 95%, a total of at least 70 subjects should be enrolled. The sample size was calculated using G*Power statistical software version 3.1.9.4 (G*Power, Heinrich Heine University, Kiel, Germany). Statistical analysis was performed applying an intention to treat analysis (last observation carried forward analysis) for the primary outcome after 12 weeks and a per‐protocol analysis for the other outcomes, using GraphPad statistical software version 5.0 (GraphPad Software, Inc., La Jolla, CA, USA). Data are expressed as mean ± SEM (standard error of the mean). A nonparametric test (Mann–Whitney test) was used for comparing treatments, a *p*‐value < 0.05 was considered as significant. An unpaired *t* test was used for the calculation of the 95% confidence interval. In addition, to compare the frequency of some results was used the chi‐square test (Fisher exact test).

## 4 RESULTS

Between May 2022 and November 2022, 83 subjects with AGA, FAGA or chronic TE were assessed and screened for inclusion in the trial. Demographics and baseline characteristic were similar between groups (Table 1). Of the 83 participants 68.7% were female, the median age was 41.2 ± 16.2, 59 subjects (71.1%) had AGA or FAGA, while 24 patients (28.9%) had chronic TE.

**TABLE 1:** Demographics and baseline characteristics.

	Total	Group A	Group B	
Patients	83 (100%)	48 (57.8%)	35 (42.2%)	
Age				
Mean (SD)	41.2 (16.2)	40.3 (17.0)	42.3 (15.3)	
Sex				
Male	26 (31.1%)	16 (33.3%)	10 (28.6%)	
Female	57 (68.7%)	32 (66.7%)	25 (71.4%)	
Hair disorder				
AGA	59 (71.1%)	33 (68.8%)	26 (74.3%)	
TE	24 (28.9%)	15 (31.3%)	9 (25.7%)	
AGA classification				
Male (n°)				
I		2	0	2
II		9	6	3
III		3	2	1
III vertex		4	2	2
IV		4	3	1
V		1	0	1
VA		1	1	0
Female (n°)				
I‐1		1	0	1
I‐2		7	5	2
I‐3		7	4	3
I‐4		12	8	4
II‐1		4	1	3
II‐2		4	1	3

**FIGURE 1:**
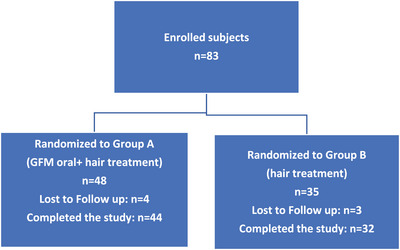
Study's flow.

Seventy‐six participants completed the trial (91.6%), and seven subjects stopped prematurely the trial for different reasons (Figure 1).

Patients, both in group A and group B received different drug treatments according to the type of hair loss. Topical Minoxidil (2% and 5%) was the treatment most used (Table 2).

**TABLE 2:** Distribution of drug treatments, alone or in combination with the food supplement used in this study, decided by the investigators according to the type of hair loss.

Treatment	% Patients
Oral finasteride	4%
Topical finasteride (0.25%)	4%
Oral minoxidil	8%
Topical minoxidil (2%)	31%
Topical minoxidil (5%)	18%
Other (e.g., supplementations, lotions, Tricopat, PRP)	24%
Not specified	11%

### 4.1 Primary endpoint

**FIGURE 2:**
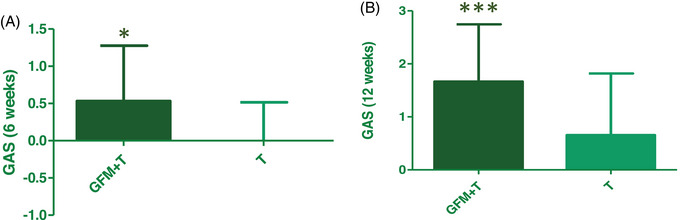
Global assessment score (GAS) evaluated after: (A) 6 weeks (group A *n* = 15, group B *n* = 16) and (B) 12 weeks (group A, *n* = 48; Group B, *n* = 35); group A: GFM+T, group B: T; *p* < 0.05, *p* < 0.001.

The GAS was evaluated in an assessor blinded fashion after 6 (facultative visit) and 12 weeks by the investigators using a 7‐point score from −3 (severe worsening) to +3 (excellent improvement). After 6 weeks, the mean GAS increased only in Group A (0.5 ± 0.2 in Group A and 0.0 ± 0.1 in group B). After 12 weeks both the two groups showed an increase in the GAS, that was higher in group A compared to group B (1.67 ± 0.16 and 0.66 ± 0.20, respectively, mean difference 1.01 ± 0.25, 95% CI: 0.52–1.50) (Figure 2). At the end of the study, the 85.4% of patients in group A showed an increase in GAS (≥0) compared to 48.6% in group B.

**FIGURE 3:**
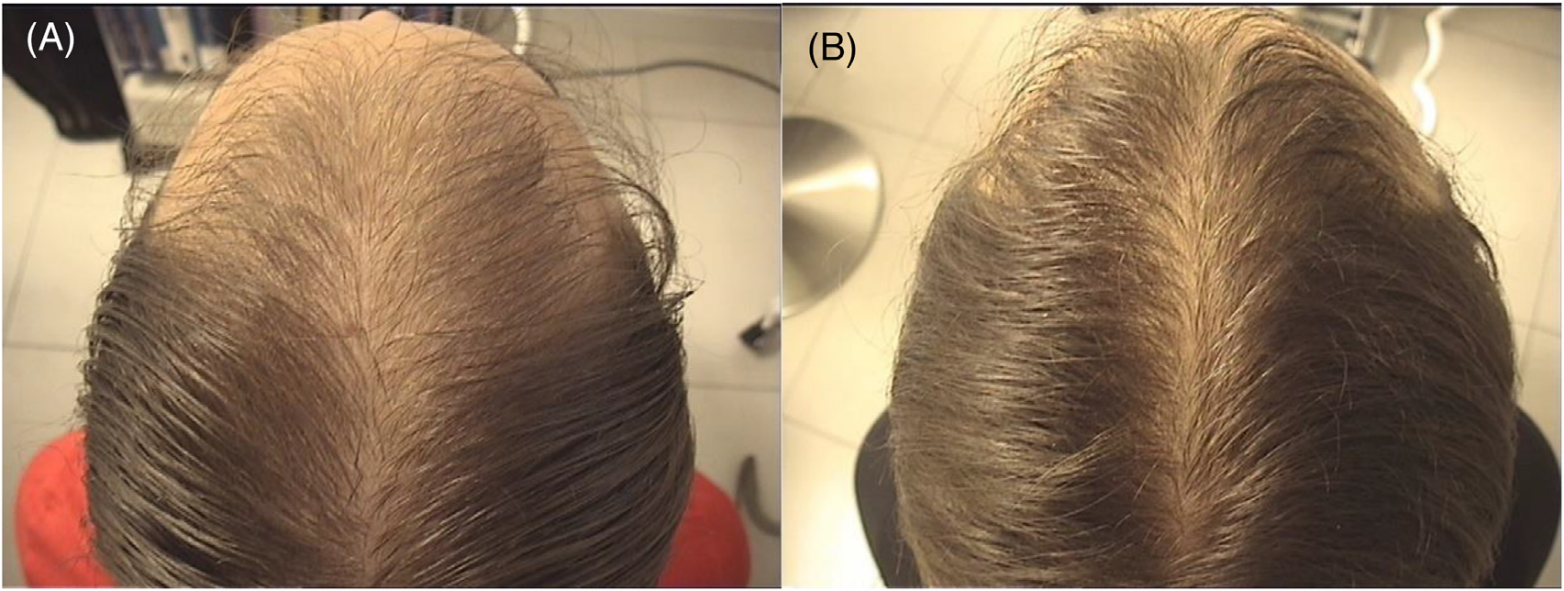
Global photographs of a patients in group A (GFM+T), took at baseline (A) and after 12 weeks (B).

**FIGURE 4:**
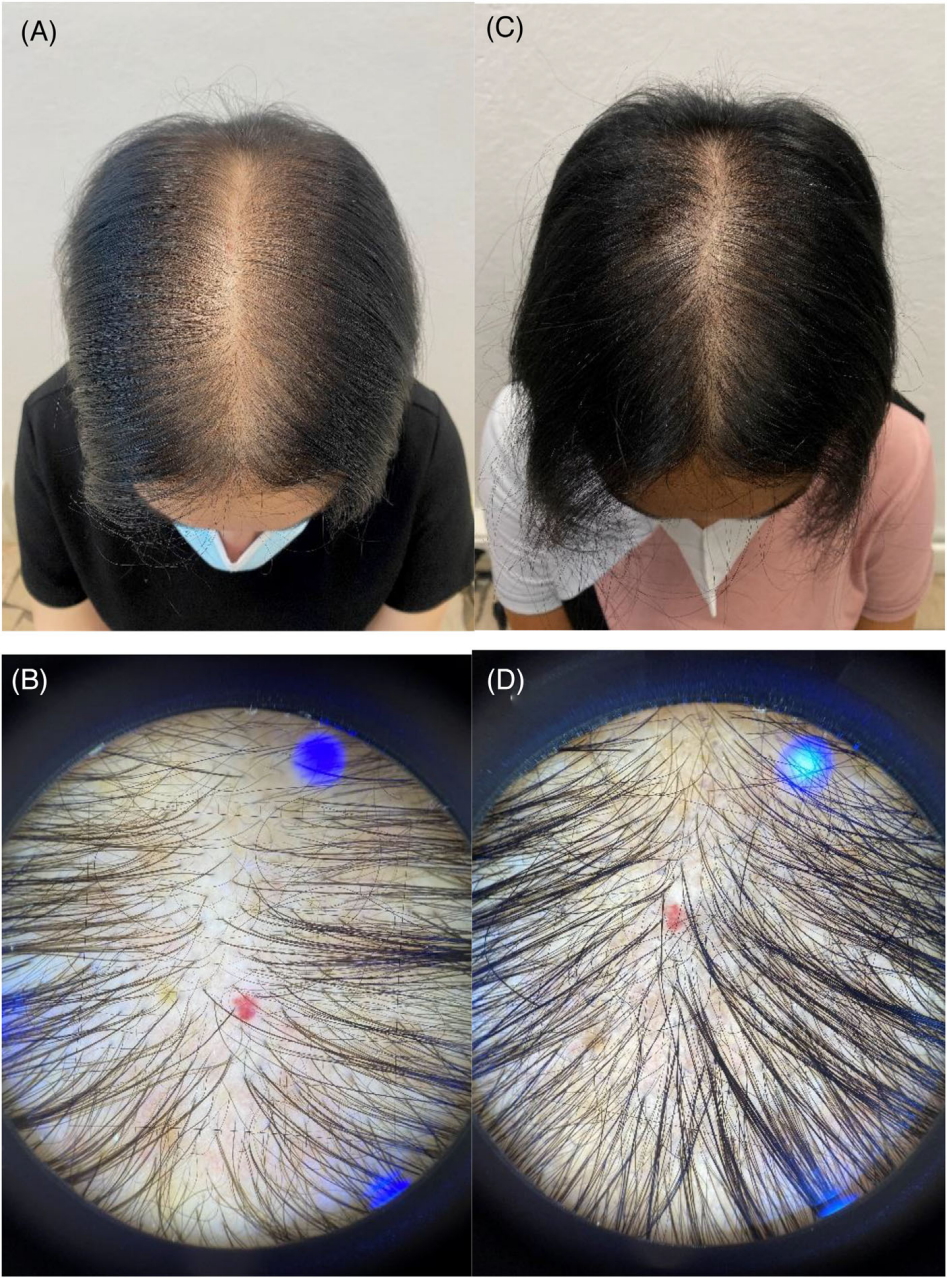
Global photographs and a macro‐photographs of a patients in group A (GFM+T), took at baseline (A and B), and after 12 weeks (C and D).

Global photographs and macro‐photographs of some patients in group A (GFM+T), took at baseline and after 12 weeks were showed in Figures 3 and 4.

### 4.2 Secondary endpoints

**FIGURE 5:**
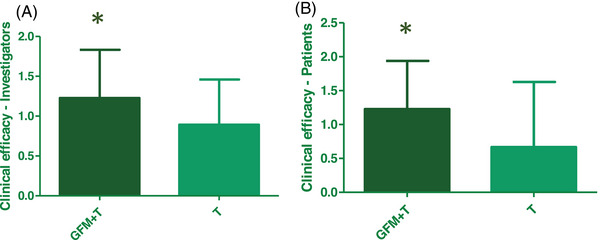
Mean score of global clinical efficacy evaluated by: (A) investigators (Group A, *n* = 44; Group B; *n* = 28) and (B) patients (Group A, *n* = 44; Group B, *n* = 27). *p < 0.05.

Global clinical efficacy was evaluated in an open fashion both by investigators and patients trough questionnaires converted in a 5‐point score scale. The mean global clinical efficacy was higher in group A both for investigators (differences between means 0.33 ± 0.14, 95% CI: 0.05–0.62) and patients (differences between means 0.56 ± 0.20, 95% CI: 0.16–0.96) (Figure 5).

In Table 3 are reported the distribution of recorded global clinical efficacy. Regarding the global clinical efficacy evaluated by investigators the frequency of “very good efficacy” in group A is statistically higher that in group B (*p* < 0.05, chi‐square test). Similarly, regarding the global clinical efficacy evaluated by patients, the frequency of “slightly improved and much improved efficacy” is statistically higher in group A compared to Group B (*p* < 0.01, chi‐square test).

**TABLE 3:** Distribution of recorded global clinical efficacy.

Investigator			Patients		
	Group A (*n* = 44)	Group B (*n* = 28)		Group A (*n* = 44)	Group B (*n* = 27)
**Very negative**	0	0	**Much worsened**	0	1 (3.7%)
**Negative**	0	0	**Slightly worsened**	0	0
**Scare**	4 (9.1%)	6 (21.4%)	**Stable**	7 (15.9%)	12 (44.4%)
**Good**	26 (59.1%)	19 (67.9%)	**Slightly improved**	20 (45.5%)	8 (29.6%)
**Very good**	14 (31.8%)	3 (10.7%)	**Much improved**	17 (38.6%)	6 (22.2%)

The tolerability of products was evaluated by investigators (Table 4) and patients (Table 5) after 12 weeks through a self‐administered questionnaire.

**TABLE 4:** Tolerability of products evaluated by investigators.

Tolerability—Investigator		
	Group A (*n* = 44)	Group B (*n* = 26)
Very negative	0	0
Negative	1 (2.3%)	0
Scare	0	1 (3.8%)
Good	23 (52.3%)	20 (76.9%)
Very good	20 (45.5%)	5 (19.2%)

**TABLE 5:** Tolerability of products evaluated by patients through a questionnaire based on a 10 items scale: From 1 (none) to 10 (a lot).

	The use/consumption of the product/s is problematic		
		Group A (*n* = 44)	Group B (*n* = 20)
None	1	37 (84.1%)	16 (80.0%)
↓	2	3 (6.8%)	2 (10.0%)
	3	0	1 (5.0%)
	4	0	0
	5	0	0
	6	2 (4.5%)	0
	7	0	1 (5.0%)
	8	1 (2.3%)	0
	9	1 (2.3%)	0
A lot	10	0	0

	Adverse gastro‐intestinal effects		
		Group A (*n* = 44)	Group B (*n* = 17)
None	1	38 (86.4%)	16 (94.1%)
↓	2	4 (9.1%)	1 (5.9%)
	3	1 (2.3%)	0
	4	0	0
	5	0	0
	6	0	0
	7	0	0
	8	0	0
	9	1 (2.3%)	0
A lot	10	0	0

Both the oral supplement and the therapies generally used to treat the hair loss conditions were well tolerated, as reported by investigators and patients. The investigators reported that the 97.7% of patients in Group A and the 96.1% in the group B showed a good or very good tolerability.

The 84.1% of subjects in group A and the 80.0% of patients in group B declare that the used of the product/s was not problematic. In addition, no gastro‐intestinal adverse effects were reported in 86.4% and 94.1% of subjects in Group A and B, respectively.

## 5 DISCUSSION

Hair loss is a common problem that affects up to 50 percent of men and women.^2^ Nutritional intervention may be beneficial for patients with hair loss. Recent evidence suggested that the used of nutritional supplementation among patients with hair loss disorders is prevalent. In a cohort study on 600 new alopecia patients, the 18.7% of subjects reported the used of oral vitamins or supplements for this condition.^20^ The most oral supplementation for hair loss included biotin (75%), vitamin B12 (11.6%), and a B‐complex multivitamin (10.7%).^20^ In addition some amino acids (like methionine, taurine, and cysteine), micronutrients and HC could be beneficial in subjects with hair loss conditions. Although for some dietary supplements clinical evidence data are available, for other ingredients clinical controlled trials are not available.^13^ Additional studies are therefore needed to fully understand the role of nutritional supplementation in manage hair loss conditions. An oral supplement containing hydrolysed fish‐origin collagen, taurine, cysteine, methionine, iron, and selenium has been recently commercialized for patients with hair loss disorders. Since no controlled data were available on the clinical efficacy of this product a prospective, 12‐week, randomized, assessor‐blinded controlled trial was conducted to test the efficacy and tolerability of this oral supplementation. Subjects included in the study were randomized to consume the oral supplementation in combination with a specify drug treatment (group A) or to use a specific drugs treatment alone (group B). The GAS was evaluated by an investigator unaware of the treatment groups at week 6 and at week 12. The GAS was statistically higher in group A compared to group B both after 6 (*p* < 0.05) and 12 weeks (*p* < 0.001), confirming the additional role of the oral supplement to improve the hair growth compared to the drug treatments alone. After 12 weeks a higher percentage of group A subjects achieved a GAS score of ≥2 in comparison with group B (50% vs. 23%). Both the oral supplement and the therapies were generally well tolerated, as reported by investigators and by patients.

Some limitations should be considered in evaluating our results. First, the two arms of the study were slightly unbalanced (48 subjects in group A and 35 subjects in group B). This could be explained by the randomization conducted using a block of six. Although the two groups are slightly unbalanced in term of participants, demographics and baseline characteristic were similar between groups. A second limitation of this study was the trial design which was not double blind. In order to increase the internal validity of our trial, we adopted an assessor‐blinded primary outcome evaluation. Another limitation of this study was related to the relatively short duration of the treatments. However, it was adequate to observe the role of oral supplementation in improving the efficacy of the anti‐hair loss treatments generally employed.

## 6 CONCLUSIONS

This clinical trial demonstrated that the tested oral supplement, containing hydrolysed fish‐origin collagen, taurine, cysteine, methionine, iron and selenium was able to improve the clinical efficacy of specific anti‐hair loss treatments in subjects with hair loss disorders such as AGA/FAGA or TE.

## CONFLICT OF INTEREST STATEMENT

MM and FC are employees of Difa Cooper Catabria Labs that commercialized the food supplement. The authors report no other conflict of interest in this work.

## ETHICS STATEMENT

All participants provided written informed consent and a photo consent statement before starting the study.

## Data Availability

The author has provided the required Data Availability Statement, and if applicable, included functional and accurate links to said data therein.
